# Trajectory Tracking Control of Transformer Inspection Robot Using Distributed Model Predictive Control

**DOI:** 10.3390/s23229238

**Published:** 2023-11-17

**Authors:** Lai Wei, Guofei Xiang, Congjun Ma, Xuejian Jiang, Songyi Dian

**Affiliations:** College of Electrical Engineering, Sichuan University, Chengdu 610065, China; weilai1@stu.scu.edu.cn (L.W.); scucongjun_ma@163.com (C.M.); 2022223035095@stu.scu.edu.cn (X.J.); scudiansy@scu.edu.cn (S.D.)

**Keywords:** underwater robot, distributed model predictive control, trajectory tracking control

## Abstract

To overcome the difficulty in tracking the trajectory of an inspection robot inside a transformer, this paper proposes a distributed model predictive control method. First, the kinematics and dynamics models of a robot in transformer oil are established based on the Lagrange equation. Then, by using the nonlinear model predictive control method and following the distributed control theory, the motion of a robot in transformer oil is decoupled into five independent subsystems. Based on this, a distributed model predictive control (DMPC) method is then developed. Finally, the simulation results indicate that a robot motion control system based on DMPC achieves high tracking accuracy and robustness with reduced computing complexity, and it provides an effective solution for the motion control of robots in narrow environments.

## 1. Introduction

Transformers play a crucial role in efficiently and economically aggregating new energy sources and constructing integrated DC transmission and distribution networks in power grids [1]. To ensure the long-term stable operation of transformers, it is necessary to perform periodic inspection and fault elimination. Currently, the maintenance of oil-immersed transformers is usually conducted through manual lifting, where specialized maintenance personnel enter the transformer’s interior to perform inspection work. To avoid humanitarian and environmental issues during an inspection process, such as during extended maintenance periods—as well as by considering the pollution concerns associated with traditional maintenance methods—robot-based transformer inspection methods have been proposed. Robot-based methods can not only perform fault detection tasks within oil-immersed transformers, but they can also avoid the aforementioned issues by using multi-vector thrusters for propulsion. Such an approach has advantages such as small size, high flexibility, and multi-degree-of-freedom (DOF) motion. Replacing manual maintenance with a transformer inspection robot can significantly improve work efficiency and cost-effectiveness.

However, the confined space inside transformers, the presence of numerous electrical components, the highly unstructured characteristics of the robot’s working environment, and the need to avoid collisions with surroundings are all great challenges for the robot’s control design. Therefore, high-performance trajectory tracking control for robots inside oil-immersed transformers is of high significance for efficient, safe, and stable underoil inspection operations. Currently, there is limited research on the trajectory tracking control of transformer inspection robots. Alongside a consideration of the similarities between the motion of robots in insulating oil and water, this paper draws inspiration from related studies on underwater robot trajectory tracking control.

Underwater robots are characterized as nonlinear control objects that are difficult to model and are underactuated, with strong coupling and susceptibility to external disturbances [2,3,4,5,6,7,8,9,10]. The primary control method for tracking control is to integrate the Lyapunov method with backstepping control. This method progressively simplifies the intricate design of the control system [2,3,4,5]. Owing to its robustness against parameter perturbations and external disturbances, sliding mode control (SMC) has emerged as another primary method for AUV trajectory tracking [6,7,8]. S. Soylu et al. [6] improved trajectory tracking performance by combining SMC, PID, and robust control methods. F. Tabataba’i-Nasab et al. [7] enhanced the robustness of AUV trajectory tracking using the sliding mode control approach, and a reasonable performance in managing the disturbances of tracking errors was achieved by exploiting a variety of terminal sliding mode tracking control (TSMTC) laws. Z. Wu et al. [8] addressed the 3D trajectory tracking problem in building an inner-loop controller that is based on nonsingular integral terminal sliding mode control (NITSMC). Neural network methods have been widely used in kinematic control systems because of their inherent nonlinearity and self-learning capabilities. By using neural networks, the nonlinearity of the underwater robot can be approximated, so there is no need to build an accurate underwater robot model. J. Zhou et al. [9] introduced a novel control algorithm, which combines the RBF neural network algorithm with state prediction by using the backstepping sliding mode control method.

However, the above control methods cannot handle multiple system constraints. The trajectory tracking process of underwater robots involves various constraints, such as the physical limitations of thrusters, the maximum allowable pitch angle of the robot’s body design, and the processing capacity of the robot’s processor. Model predictive control (MPC) can effectively handle multiple constraints, thereby providing robust support for solving various constraint control problems [10,11,12,13,14,15,16,17,18,19,20]. Y. Chen et al. [11] proposed a tube-based MPC to handle the kinematic constraints for nonholonomic mobile robot tracking control. J. Zhang et al. [12] designed an MPC controller that incorporates Lyapunov-based input constraints to address the trajectory tracking problem in nonholonomic wheeled mobile robots. S. Liu et al. [13] presented a hierarchical control strategy consisting of a local layer that was based on a model of predictive control, which effectively addressed both tracking problems and mixed constraints on physical states simultaneously. MPC is suitable for complex nonlinear systems, thus making it one of the mainstream methods for underwater robot trajectory tracking control [21,22,23,24,25,26,27,28,29,30]. C. Shen et al. [30] established an innovative Lyapunov-based model predictive control framework for the AUV by leveraging computational resources to improve the trajectory tracking performance. Z. Yan et al. [22] designed a double closed-loop trajectory tracking method based on model predictive control by considering the actual constraints on the system inputs and state. P. Gong et al. [23] designed a tracking controller that is based on a dual closed-loop of MPC that incorporates backstepping control into an inner-loop controller to keep the system stable. Z. Yan et al. [25] proposed a robust model predictive control method to handle the trajectory tracking of AUV, as well as designed a finite-time extended state observer to compensate for dynamic model uncertainty.

Although MPC effectively addresses the issue of nonlinear dynamic constraints, it also brings about a great computational challenge. In the case of nonlinear underwater robots, the computational complexity increases exponentially with the number of control variables. Distributed model predictive control (DMPC) defines optimization objectives for each subsystem, and it independently solves optimization problems between the subsystems instead of centralizing the computation of a global optimization problem. This method significantly reduces the computational burden on processors. In addition, DMPC divides the single online optimization problem of complex large-scale systems into multiple sub-problems for parallel processing, which greatly reduces the size and complexity of individual optimization problems, as well as accelerates the computation speed of the global system optimization problem, thereby ensuring real-time system performance. Compared to centralized model predictive control, DMPC can achieve global coordination through local information; thus, it greatly improves the real-time nature of control [31]. It has been widely used in the field of ground vehicles [32,33,34,35]. By using DMPC to control AUV motion, Y. Bian et al. [34] divided the system into two subsystems for the depth and longitudinal velocity control, as well as sacrificed some depth control convergence speed to reduce most of the computation time.

This paper proposes a trajectory tracking control method based on distributed model predictive control. The motion of the robot in transformer oil is firstly decoupled into five independent subsystems, which are then transformed into predictive optimization problems to realize a high-precision tracking of the given trajectory by the transformer inspection robot within the oil-immersed transformer. The main contributions of this paper are summarized as follows:

(1) A DMPC algorithm is proposed to solve the transformer inspection robot trajectory tracking control problem. With the proposed DMPC, the performance and robustness of the tracking control can be improved.
(2) Through the DMPC method, by decoupling the transformer inspection robot’s motion into five subsystems, the computational complexity of the trajectory tracking control can be reduced while the tracking performance can be maintained.
(3) We validate the proposed method in a variety of simulations. The simulations aim to demonstrate the advantages of the DMPC tracking method compared with some other baseline methods.

The rest of this paper can be summarized as follows: Section 2 describes the kinematics and dynamics models of a robot in transformer oil based on the Lagrange equation. In Section 3, the trajectory tracking problem of the transformer inspection robot is formulated, and the difficulties in solving this problem are analyzed. In Section 4, based on the nonlinear model predictive control method and distributed control theory, a DMPC algorithm is designed to solve the problem. Section 5 presents the simulation results of the trajectory tracking. Finally, Section 6 concludes the paper.

## 2. System Modeling

**Figure 1:**
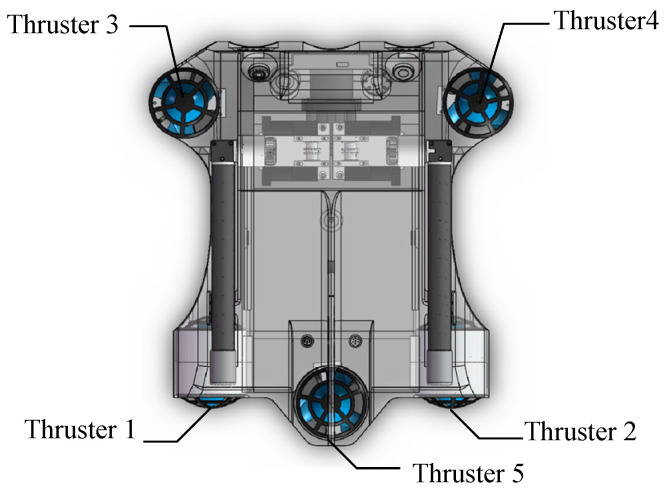
Structural diagram of the transformer inspection robot.

The structural diagram of the transformer inspection robot studied in this paper is illustrated in Figure 1. The robot’s propulsion system consists of five thrusters. Specifically, two horizontal thrusters are symmetrically distributed along the axis to enable the robot to have two degrees of freedom for forward and backward motion, as well as yaw motion. Meanwhile, three vertical thrusters are symmetrically arranged in an equilateral triangle along the axis, thus enabling the robot to have three degrees of freedom for ascending/descending, rolling, and pitching motions.

According to the arrangement of the robot’s thrusters, the thrust allocation for each thruster under different operating conditions is listed in Table 1.

### 2.1. Kinematic Model

The motion of a robot in transformer oil is highly complex and exhibits nonlinear characteristics. To simplify this problem, the following assumptions are made in this paper:

**Assumption 1:** When the robot is moving only on the vertical plane, its heading and longitudinal velocity remain constant, i.e., the center of gravity of the robot remains on the vertical plane during vertical motion.

**Assumption 2:** As the robot changes its heading and longitudinal velocity, its depth remains constant. Consequently, the center of gravity of the robot remains on the horizontal plane during horizontal motion.

**Assumption 3:** As the robot’s orientation changes, its depth and longitudinal velocity remain consistent. The position of the robot’s center of gravity remains unchanged.

**Figure 2:**
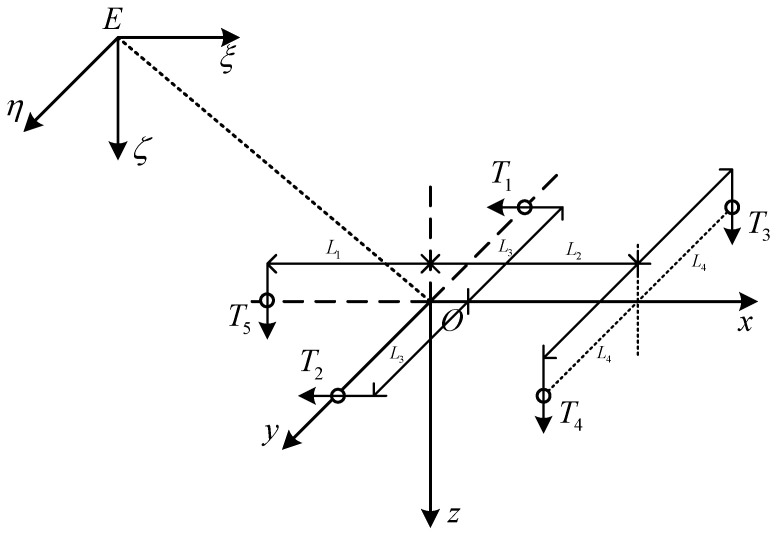
The robot inertial coordinate system and body-fixed coordinate system.

Considering the motion characteristics of a robot inside an oil-immersed transformer, a coordinate system for the robot’s motion is established in Figure 2. This coordinate system includes the inertial coordinate system E−ξηζ and the body-fixed coordinate system O−xyz. The origin of the body-fixed coordinate system coincides with the center of gravity of the robot.

The robot’s position coordinates in the inertial coordinate system are represented as (x,y,z), and its orientation angles are (φ,θ,ψ). Here, *x* denotes the longitudinal coordinate, *y* denotes the lateral coordinate, *z* denotes the vertical coordinate, φ denotes the roll angle, θ denotes the pitch angle, and ψ denotes the yaw angle of the robot in the inertial coordinate system. The linear velocity of the robot in the body-fixed coordinate system is represented as (u,v,w), and the angular velocity is represented as (p,q,r). The kinematics model of the robot can be expressed as follows:

(1) $$\dot{\eta }=J\left(\eta \right)\nu$$

where $\eta ={\left[x,y,z,\varphi ,\theta ,\psi \right]}^{T}$ denotes the position and pose of the robot in the inertial coordinate system. $\nu ={\left[u,v,w,p,q,r\right]}^{T}$ denotes the velocity and angular velocity of the robot in the body-fixed coordinate system. J(η) represents the conversion matrix, and it is defined as follows:

(2) $$J\left(\eta \right)=\left[\begin{array}{cc} J_{1}\left(\eta \right) & O_{3\times 3}\\ O_{3\times 3} & J_{2}\left(\eta \right) \end{array}\right]$$

Regarding linear velocities, the conversion matrix $J_{1}\left(\eta \right)$ from the body-fixed coordinate system to the inertial coordinate system is given by

(3) $$J_{1}\left(\eta \right)=\left[\begin{array}{ccc} c\psi c\theta & -s\psi c\varphi +c\psi s\varphi s\theta & s\psi s\varphi +c\psi c\varphi s\theta \\ s\psi c\theta & c\psi c\varphi +s\psi s\varphi s\theta & -c\psi s\varphi +s\psi c\varphi s\theta \\ -s\theta & c\theta s\varphi & c\theta c\varphi \end{array}\right]$$

Regarding angular velocities, the conversion matrix $J_{2}\left(\eta \right)$ from the body-fixed coordinate system to the inertial coordinate system is given by

(4) $$J_{2}\left(\eta \right)=\left[\begin{array}{ccc} 1 & s\varphi t\theta & c\varphi t\theta \\ 0 & c\varphi & -s\varphi \\ 0 & s\varphi /c\theta & c\varphi /c\theta \end{array}\right]$$

where *c*, *s*, and *t* denote the shorthand of cos(·), sin(·), and tan(·), respectively.

Taking into account the motion of the robot in the real world, the roll angle φ needs to satisfy |φ|<π/2. Moreover, to avoid singularities, the pitch angle θ needs to satisfy |θ|<π/2. Substituting (2)–(4) into (1) yields the following kinematics model for the robot’s six degrees of freedom:

(5) $$\left\{\begin{array}{c} \dot{x}=uc\psi c\theta +v\left(-s\psi c\varphi +c\psi s\varphi s\theta \right)+w\left(s\psi s\varphi +c\psi c\varphi s\theta \right)\\ \dot{y}=us\psi c\theta +v\left(c\psi c\varphi +s\psi s\varphi s\theta \right)+w\left(-c\psi s\varphi +s\psi c\varphi s\theta \right)\\ \dot{z}=-us\theta +vc\theta s\varphi +wc\theta c\varphi \\ \dot{\varphi }=p+qs\varphi t\theta +rc\varphi t\theta \\ \dot{\theta }=qc\varphi -rs\varphi \\ \dot{\psi }=q\left(s\varphi /c\theta \right)+r\left(c\varphi /c\theta \right) \end{array}\right.$$

### 2.2. Dynamic Model

The conventional 6-DOF dynamic model for underwater robots is described as follows:

(6) $$M\dot{\nu }+C\left(\nu \right)\nu +D\left(\nu \right)\nu +G\left(\eta \right)=\tau$$

where $M=M_{RB}+M_{A}$, in which $M_{RB}$ and $M_{A}$ represent the body mass inertia matrix and the additional mass inertia matrix, respectively. $C\left(\nu \right)=C_{RB}\left(\nu \right)+C_{A}\left(\nu \right)$, in which $C_{RB}$ represents the Coriolis and centripetal force matrix, and $C_{A}$ represents the additional mass generated due to the Coriolis and centripetal forces. D(ν) is the damping matrix, G(η) represents the restoring force, and τ represents the propulsion forces and moments. Thus, by expanding the dynamic equations, we have

(7) $$\left\{\begin{array}{c} F_{u}=\left(m-X_{\dot{u}}\right)\dot{u}+\left(m-Z_{\dot{w}}\right)wq+\left(-m+Y_{\dot{v}}\right)vr-\left(X_{u}+X_{u|u|}\left|u\right|\right)u-\left(H-W\right)s\theta \\ 0=\left(m-Y_{\dot{v}}\right)\dot{v}+\left(-m+Z_{\dot{w}}\right)wp+\left(m-X_{\dot{u}}\right)ur-\left(Y_{v}+Y_{v|v|}\left|v\right|\right)v+\left(H-W\right)c\theta s\varphi \\ F_{w}=\left(m-Z_{\dot{w}}\right)\dot{w}+\left(m-Y_{\dot{v}}\right)vp+\left(-m+X_{\dot{u}}\right)uq-\left(Z_{w}+Z_{w|w|}\left|w\right|\right)w-\left(H-W\right)c\theta c\varphi \\ F_{p}=\left(I_{x}-K_{\dot{p}}\right)\dot{p}+\left(Y_{\dot{v}}-Z_{\dot{w}}\right)vw+\left(I_{z}-N_{\dot{r}}-I_{y}+M_{\dot{q}}\right)qr-\left(K_{p}+K_{p|p|}\left|p\right|\right)p+z_{B}Hs\varphi c\theta \\ F_{q}=\left(I_{y}-M_{\dot{q}}\right)\dot{q}+\left(Z_{\dot{w}}-X_{\dot{u}}\right)uw+\left(-I_{z}+N_{\dot{r}}+I_{x}-K_{\dot{p}}\right)pr-\left(M_{q}+M_{q|q|}\left|q\right|\right)q+z_{B}Hs\theta \\ F_{r}=\left(I_{z}-N_{\dot{r}}\right)\dot{r}+\left(X_{\dot{u}}-Y_{\dot{v}}\right)uv+\left(I_{y}-M_{\dot{q}}-I_{x}+K_{\dot{p}}\right)pq-\left(N_{r}+N_{r|r|}\left|r\right|\right)r \end{array}\right.$$

where *m* is the mass of the robot; $X_{\dot{u}},Y_{\dot{v}},Z_{\dot{w}},K_{\dot{p}},M_{\dot{q}},$ and $N_{\dot{r}}$ are the additional mass terms; $I_{x},I_{y},$ and $I_{z}$ are the inertia tensors; $X_{u}$, $Y_{v}$, $Z_{w}$, $K_{p}$, $M_{q}$, $N_{r}$, $X_{u|u|}\left|u\right|$, $Y_{v|v|}\left|v\right|$, $Z_{w|w|}\left|w\right|$, $K_{p|p|}\left|p\right|$, $M_{q|q|}\left|q\right|$, and $N_{r|r|}\left|r\right|$ are the linear damping and quadratic damping terms; *H* is the buoyancy of the robot; and *W* is the gravity of the robot. Furthermore, the origin of the motion coordinate system coincides with the center of gravity of the robot, and the center of buoyancy of the robot is slightly higher than the center of gravity. The actual position of the center of gravity in the motion coordinate system is [0,0,0], and the actual position of the center of buoyancy is $\left[0,0,z_{B}\right]$.

The generalized thrust force $\tau ={\left[F_{u},F_{v},F_{w},F_{p},F_{q},F_{r}\right]}^{T}$ is generated by five thrusters $u={\left[T_{1},T_{2},T_{3},T_{4},T_{5}\right]}^{T}$, which follow τ=Bu, where *B* is a constant input matrix in the following form:

(8) $$B=\left[\begin{array}{ccccc} 1 & 1 & 0 & 0 & 0\\ 0 & 0 & 0 & 0 & 0\\ 0 & 0 & -1 & -1 & -2L_{2}/L_{1}\\ 0 & 0 & L_{4} & -L_{4} & 0\\ 0 & 0 & L_{2} & L_{2} & -L_{1}\\ L_{3} & -L_{3} & 0 & 0 & 0 \end{array}\right]$$

where $L_{1}$, $L_{2}$, $L_{3}$, and $L_{4}$ is the linear distance from the center of each thruster to the center of the robot.

The model used for the design of the robot trajectory tracking control is defined as follows:

(9) $$\begin{array}{cc} \dot{x}\; & =\left[\begin{array}{c} J(\eta )v\\ M^{-1}(Bu-C\left(v\right)-D\left(v\right)-G\left(\eta \right) \end{array}\right]\\ \; & ≜f(x,u) \end{array}$$

where $x={\left[x,y,z,\varphi ,\theta ,\psi ,u,v,w,p,q,r\right]}^{T}$ denotes the system state, and the control is defined as $u={\left[T_{1},T_{2},T_{3},T_{4},T_{5}\right]}^{T}$.

## 3. Problem Formulation

This study addresses the trajectory tracking problem of a transformer inspection robot. The objective is to guarantee that the robot’s pose and state can follow a predefined reference trajectory, which is represented in a parameterized form. The reference trajectory is defined as follows:

(10) $$\rho \left(t\right)=[x_{R}\left(t\right),y_{R}\left(t\right),z_{R}\left(t\right)]$$

**Assumption 4:** The desired trajectory ρ(t) and its derivatives are smooth, bounded, and satisfy $0\leq \rho \leq {∥\rho \left(t\right)∥}_{\infty }\leq \bar{\rho }<\infty$, $0\leq {\underline{\rho }}_{1}\leq {∥\dot{\rho }\left(t\right)∥}_{\infty }\leq {\bar{\rho }}_{1}<\infty$, $0\leq {\underline{\rho }}_{2}\leq {∥\ddot{\rho }\left(t\right)∥}_{\infty }\leq {\bar{\rho }}_{2}<\infty$, $0\leq \rho_{3}\leq {∥\left(t\right)∥}_{\infty }\leq {\bar{\rho }}_{3}<\infty$.

Regarding the reference trajectory, each point on it is considered to have the same kinematic characteristics as the robot’s state trajectory. Let $x_{R}\left(t\right)={\left[x_{R}\left(t\right),y_{R}\left(t\right),z_{R}\left(t\right),\varphi_{R}\left(t\right),\theta_{R}\left(t\right),\psi_{R}\left(t\right),u_{R}\left(t\right),v_{R}\left(t\right),w_{R}\left(t\right),p_{R}\left(t\right),q_{R}\left(t\right),r_{R}\left(t\right)\right]}^{T}$ with

(11) $$\begin{array}{cc} \; & \varphi_{R}\left(t\right)=0,\;\theta_{R}\left(t\right)=-atan2\left({\dot{z}}_{R},\sqrt{{\dot{x}}_{R}^{2}+{\dot{y}}_{R}^{2}}\right),\;\psi_{R}\left(t\right)=atan2\left({\dot{y}}_{R},{\dot{x}}_{R}\right)\\ \; & u_{R}\left(t\right)=\sqrt{{\dot{x}}_{R}^{2}+{\dot{y}}_{R}^{2}},\;v_{R}\left(t\right)=0,\;w_{R}\left(t\right)={\dot{z}}_{R}\\ \; & p_{R}\left(t\right)=0,\;q_{R}\left(t\right)=\left({\dot{z}}_{R}{\ddot{x}}_{R}-{\dot{x}}_{R}{\ddot{z}}_{R}\right)/\left({\dot{z}}_{R}^{2}+{\dot{x}}_{R}^{2}\right),\;r_{R}\left(t\right)=\left({\dot{x}}_{R}{\ddot{y}}_{R}-{\dot{y}}_{R}{\ddot{x}}_{R}\right)/\left({\dot{x}}_{R}^{2}+{\dot{y}}_{R}^{2}\right), \end{array}$$

where atan2 denotes the four-quadrant inverse tangent operator. It can be confirmed that $x_{R}\left(t\right)$ complies with the kinematics model in (1). The reference control forces $\tau_{R}={\left[F_{uR},F_{vR},F_{wR},F_{pR},F_{qR},F_{rR}\right]}^{T}$ can be obtained by

(12) $$\tau_{R}=M{\dot{v}}_{R}+C\left(v_{R}\right)v_{R}+D\left(v_{R}\right)v_{R}+G\left(\eta_{R}\right)$$

where $\eta_{R}={\left[x_{R}\left(t\right),y_{R}\left(t\right),z_{R}\left(t\right),\varphi_{R}\left(t\right),\theta_{R}\left(t\right),\psi_{R}\left(t\right)\right]}^{T}$, $\nu_{R}={\left[u_{R}\left(t\right),v_{R}\left(t\right),w_{R}\left(t\right),p_{R}\left(t\right),q_{R}\left(t\right),r_{R}\left(t\right)\right]}^{T}$. Each state of the robot has a reference.

The formulation of the MPC for the robot trajectory tracking control can be defined as follows:

(13) $$\begin{array}{c} \min_{\hat{u}\left(s,t_{0}\right)}J=\int_{0}^{T}∥\hat{x}\left(s,t_{0}\right)-x_{R}\left(t_{0}+s\right){∥}_{Q}^{2}+∥\hat{u}\left(s,t_{0}\right){∥}_{R}^{2}ds+{∥\hat{x}\left(T,t_{0}\right)-x_{R}\left(t_{0}+T\right)∥}_{P}^{2}\\ \begin{array}{cc} s.t.\; & \dot{\hat{x}}\left(s,t_{0}\right)=f\left(\hat{x}\left(s,t_{0}\right),\hat{u}\left(s,t_{0}\right)\right)\\ & \hat{x}\left(0,t_{0}\right)=x\left(t_{0}\right)\\ & u_{\min}\leq \hat{u}\left(s,t_{0}\right)\leq u_{\max} \end{array} \end{array}$$

where $\hat{x}\left(s,t_{0}\right)$ denotes the predicted state trajectory of the robot evolving from $t_{0}$; $\hat{u}\left(s\right)$ denotes the predictive control; $\hat{x}-x_{R}$ denotes the error state; S(δ) denotes the family of the piecewise constant functions that are characterized by the sampling period δ; T=Nδ denotes the prediction horizon; and Q,R,P are the weighting matrices, which are positive definite.

To discretize (13), we used the following:

(14) $$\begin{array}{c} \min_{U}J_{d}\left(U,x\right)=\sum_{k=0}^{N-1}(∥x_{k}-x_{R,k}{∥}_{Q}^{2}+∥u_{k}{∥}_{R}^{2})+∥x_{k}-x_{R,N}{∥}_{P}^{2}\\ \begin{array}{cc} s.t.\; & x_{k+1}=f_{d}\left(x_{k},u_{k}\right)\\ & x_{0}=x\\ & u_{\min}\leq u_{k}\leq u_{\max} \end{array} \end{array}$$

where, the system model is discretized and represented by $x_{k+1}=f_{d}\left(x_{k},u_{k}\right)$ with $x_{i}=x\left(t+i\Delta t\right)$ and $u_{i}=u\left(t+i\Delta t\right)$. *U* denotes the sequence of the control inputs, and they are the decision variables in the optimization problem.

Generally, $f_{d}$ is highly nonlinear and non-convex, so solving (14) is a nonlinear programming (NLP) problem, which is extremely difficult to solve efficiently. NLP problems require solving the Hamilton–Jacobi–Bellman (HJB) equation. However, for constrained nonlinear systems, it is not feasible to obtain the exact analytical solution of the system’s HJB equation directly. Therefore, numerical methods are needed for solving such problems. Sequential quadratic programming (SQP) is a widely used method for resolving constrained nonlinear programming problems. The computational complexity of the SQP method is directly related to the dimensionality of the control inputs. If the dimensionality is high, the size of the subproblems will also increase, thus leading to increased computational costs for solving the subproblems.

Generally, the expected flop count is employed to estimate the computational complexity of the SQP, and it is obtained as

(15) $$\#\;\mathrm{flops}{\;}_{SQP}=i_{SQP}\times i_{Ip}\left(2/3{\left(Nm\right)}^{3}+2{\left(Nm\right)}^{2}\right)$$

where $i_{SQP}$ denotes the number of SQP iterations, $i_{Ip}$ denotes the number of interior point iterations, *N* denotes the prediction horizon, and *m* denotes the number of control inputs.

The computational complexity involved in solving Equation (14) is extremely high because it grows exponentially with the number of control inputs, which is impractical for real-time on-board implementation. In addition, it can be seen from Equation (15) that reducing the number of control inputs *m* can lower the computational complexity. Thus, this study reduced the number of control inputs from 5 to 1, and the flop count was also reduced by 95.90%. This observation motivated us in this study to use a distributed method for solving the MPC problem.

## 4. Distributed Model Predictive Control Algorithm Design

To simplify the distributed implementation, the thrust allocation (TA) from the calculation of the tracking control law was utilized. If $B^{T}B$ is nonsingular, the following Moore–Penrose pseudoinverse method can be utilized as the closed-form solution for the TA:

(16) $$u={\left(B^{T}B\right)}^{-1}B^{T}\tau =B^{*}\tau$$

Then, we selected $\tau ={\left[F_{u},F_{v},F_{w},F_{p},F_{q},F_{r}\right]}^{T}$ as the decision variables in Equation (13). Instead of substituting $u_{\min}\leq \left|B^{*}\tau \left(s,t_{0}\right)\right|\leq u_{\max}$ for $u_{\min}\leq u\left(s,t_{0}\right)\leq u_{\max}$, we employed $\tau_{\min}\leq \left|\tau \left(s,t_{0}\right)\right|\leq \tau_{\max}$, which imposes a direct constraint on the decision variables.

The transformer inspection robot is a nonlinear and strongly coupled system, with motion between any of the degrees of freedom that exhibit coupling relationships. When designing the motion controller for the robot, the degree of coupling between the different DOFs should be minimized. This can be achieved by independently designing controllers for the multiple DOFs, as well as by adopting a distributed cooperative control method instead of a centralized controller with thrust allocation control. When controllers on each DOF work independently, it reduces the difficulty due to the motion coupling in the design of a centralized controller. Additionally, enhancing the performance of controllers on each DOF can enhance the overall control effectiveness of a robot.

To simplify the design of motion control according to the characters of robot motion, the 6-DOF nonlinear Equations (1) and (6) of the robot can be decoupled into three subsystems: the surge speed control subsystem u(t), the steering control subsystem (v(t),r(t),ψ(t)), and the diving control subsystem (w(t),p(t),q(t),z(t),ϕ(t),θ(t)).

From Equations (7) and (11), it can be observed that, along the desired reference trajectory, the dynamic equations governing the robot’s motion exhibit loose coupling. In fact, since v=p=0 in the steering control subsystem, the sway dynamics and yaw dynamics are decoupled; in the diving control system, the heave dynamics, roll dynamics, and pitch dynamics are decoupled. Meanwhile, it can be seen from Equation (8) that the robot is an underactuated system that lacks thrusters for control in the sway direction. From this, the motion of the transformer inspection robot can be divided into five subsystems: the surge subsystem, the yaw subsystem, the heave subsystem, the roll subsystem, and the pitch subsystem. Within the framework of distributed control, the input for trajectory tracking is computed in parallel by these five subsystems.

The surge subsystem model is represented as

(17) $$\dot{\xi }\left(s\right)=f_{1}\left(\xi \left(s\right),\hat{v}\left(s\right),\hat{w}\left(s\right),\hat{p}\left(s\right),\hat{q}\left(s\right),\hat{r}\left(s\right),F_{u}\left(s\right)\right)$$

with the state $\xi ={\left[x,y,z,\phi ,\theta ,\psi ,u\right]}^{T}$. The surge subsystem exclusively focuses on the surge dynamics and kinematics. The control input $F_{u}$ is determined by the cost function $J_{1}$ as follows:

(18) $$\begin{array}{cc} \; & \min_{F_{u}\in S\left(s\right)}J_{1}=\int_{0}^{T}(∥\tilde{x}{\left(s\right)∥}_{Q}^{2}+r_{11}F_{u}^{2}\left(s\right))ds+∥\tilde{x}{\left(T\right)∥}_{P}^{2}\\ \; & \begin{array}{cc} s.t.\; & \dot{\xi }\left(s\right)=f_{1}\left(\xi \left(s\right),\hat{v}\left(s\right),\hat{w}\left(s\right),\hat{p}\left(s\right),\hat{q}\left(s\right),\hat{r}\left(s\right),F_{u}\left(s\right)\right)\\ \; & \xi \left(0\right)=\xi \left(t_{0}\right)\\ \; & |F_{u}\left(s\right)|\leq F_{u,\max} \end{array} \end{array}$$

where $\hat{v}\left(s\right),\hat{w}\left(s\right),\hat{p}\left(s\right),\hat{q}\left(s\right),$ and $\hat{r}\left(s\right)$ are considered as the known coefficients when optimizing and solving for the individual subsystems.

The heave subsystem model is represented by

(19) $$\dot{\chi }\left(s\right)=f_{2}\left(\chi \left(s\right),\hat{u}\left(s\right),\hat{v}\left(s\right),\hat{p}\left(s\right),\hat{q}\left(s\right),\hat{r}\left(s\right),F_{w}\left(s\right)\right)$$

with the state $\chi ={\left[x,y,z,\phi ,\theta ,\psi ,w\right]}^{T}$. The heave subsystem only considers the heave dynamics and kinematics. The control input $F_{w}$ is determined by the cost function $J_{2}$ as follows:

(20) $$\begin{array}{cc} \; & \min_{F_{w}\in S\left(\delta \right)}J_{2}=\int_{0}^{T}(∥\tilde{x}{\left(s\right)∥}_{Q}^{2}+r_{22}F_{w}^{2}\left(s\right))ds+∥\tilde{x}{\left(T\right)∥}_{P}^{2}\\ \; & \begin{array}{cc} s.t.\; & \dot{\chi }\left(s\right)=f_{2}\left(\chi \left(s\right),\hat{u}\left(s\right),\hat{v}\left(s\right),\hat{p}\left(s\right),\hat{q}\left(s\right),\hat{r}\left(s\right),F_{w}\left(s\right)\right)\\ \; & \chi \left(0\right)=\chi \left(t_{0}\right)\\ \; & |F_{w}\left(s\right)|\leq F_{w,\max} \end{array} \end{array}$$

where $\hat{u}\left(s\right),\hat{v}\left(s\right),\hat{p}\left(s\right),\hat{q}\left(s\right),$ and $\hat{r}\left(s\right)$ are considered the known coefficients when optimizing and solving the individual subsystems.

The roll subsystem model is represented as

(21) $$\dot{\sigma }\left(s\right)=f_{3}\left(\sigma \left(s\right),\hat{u}\left(s\right),\hat{v}\left(s\right),\hat{w}\left(s\right),\hat{q}\left(s\right),\hat{r}\left(s\right),F_{p}\left(s\right)\right)$$

with the state $\sigma ={\left[x,y,z,\phi ,\theta ,\psi ,p\right]}^{T}$. The roll subsystem exclusively handles the roll dynamics and kinematics. The cost function $J_{3}$ is determined by the control input $F_{p}$ as follows:

(22) $$\begin{array}{cc} \; & \min_{F_{p}\in S\left(\delta \right)}J_{3}=\int_{0}^{T}(∥\tilde{x}{\left(s\right)∥}_{\underline{Q}}^{2}+r_{33}F_{p}^{2}\left(s\right))ds+∥\tilde{x}{\left(T\right)∥}_{P}^{2}\\ \; & \begin{array}{cc} s.t.\; & \dot{\sigma }\left(s\right)=f_{3}\left(\sigma \left(s\right),\hat{u}\left(s\right),\hat{v}\left(s\right),\hat{w}\left(s\right),\hat{q}\left(s\right),\hat{r}\left(s\right),F_{p}\left(s\right)\right)\\ \; & \sigma \left(0\right)=\sigma \left(t_{0}\right)\\ \; & |F_{p}\left(s\right)|\leq F_{p,\max} \end{array} \end{array}$$

where $\hat{u}\left(s\right),\hat{v}\left(s\right),\hat{w}\left(s\right),\hat{q}\left(s\right),$ and $\hat{r}\left(s\right)$ are considered the known coefficients when optimizing and solving the individual subsystems.

The pitch subsystem model is represented as

(23) $$\dot{\kappa }\left(s\right)=f_{4}\left(\kappa \left(s\right),\hat{u}\left(s\right),\hat{v}\left(s\right),\hat{w}\left(s\right),\hat{p}\left(s\right),\hat{r}\left(s\right),F_{q}\left(s\right)\right)$$

with the state $\kappa ={\left[x,y,z,\phi ,\theta ,\psi ,q\right]}^{T}$. The pitch subsystem exclusively handles the pitch dynamics and kinematics. The cost function $J_{4}$ is determined by the control input $F_{q}$ as follows:

(24) $$\begin{array}{cc} \; & \min_{F_{q}\in S\left(\delta \right)}J_{4}=\int_{0}^{T}(∥\tilde{x}{\left(s\right)∥}_{Q}^{2}+r_{44}F_{q}^{2}\left(s\right))ds+∥\tilde{x}{\left(T\right)∥}_{P}^{2}\\ \; & \begin{array}{cc} s.t.\; & \dot{\kappa }\left(s\right)=f_{4}\left(\kappa \left(s\right),\hat{u}\left(s\right),\hat{v}\left(s\right),\hat{w}\left(s\right),\hat{p}\left(s\right),\hat{r}\left(s\right),F_{q}\left(s\right)\right)\\ \; & \kappa \left(0\right)=\kappa \left(t_{0}\right)\\ \; & |F_{q}\left(s\right)|\leq F_{q,\max} \end{array} \end{array}$$

where $\hat{u}\left(s\right),\hat{v}\left(s\right),\hat{w}\left(s\right),\hat{p}\left(s\right),$ and $\hat{r}\left(s\right)$ are considered the known coefficients when optimizing and solving the individual subsystems.

The yaw subsystem model is represented by

(25) $$\dot{\lambda }\left(s\right)=f_{5}\left(\lambda \left(s\right),\hat{u}\left(s\right),\hat{v}\left(s\right),\hat{w}\left(s\right),\hat{p}\left(s\right),\hat{q}\left(s\right),F_{r}\left(s\right)\right)$$

with the state $\lambda ={\left[x,y,z,\phi ,\theta ,\psi ,r\right]}^{T}$. The yaw subsystem exclusively handles the yaw dynamics and kinematics. The cost function $J_{5}$ is determined by the control input $F_{r}$ as follows:

(26) $$\begin{array}{cc} \; & \min_{F_{r}\in S\left(\delta \right)}J_{5}=\int_{0}^{T}(∥\tilde{x}{\left(s\right)∥}_{Q}^{2}+r_{55}F_{p}^{2}\left(s\right))ds+∥\tilde{x}{\left(T\right)∥}_{P}^{2}\\ \; & \begin{array}{cc} s.t.\; & \dot{\lambda }\left(s\right)=f_{5}\left(\lambda \left(s\right),\hat{u}\left(s\right),\hat{v}\left(s\right),\hat{w}\left(s\right),\hat{p}\left(s\right),\hat{q}\left(s\right),F_{r}\left(s\right)\right)\\ \; & \lambda \left(0\right)=\lambda \left(t_{0}\right)\\ \; & |F_{r}\left(s\right)|\leq F_{r,\max} \end{array} \end{array}$$

where $\hat{u}\left(s\right),\hat{v}\left(s\right),\hat{w}\left(s\right),\hat{p}\left(s\right),$ and $\hat{q}\left(s\right)$ are considered the known coefficients when optimizing and solving the individual subsystems.

The process of determining the assumed state trajectories $\hat{u}\left(s\right),\hat{v}\left(s\right),\hat{w}\left(s\right),\hat{p}\left(s\right),\hat{q}\left(s\right),$ and $\hat{r}\left(s\right)$ can be outlined as follows: First, consider $F_{u}^{*},F_{w}^{*},F_{p}^{*},F_{q}^{*},$ and $F_{r}^{*}$ as the optimal solutions to the subproblems (18), (20), (22), (24), and (26) at the previous time. Then, at the current time, use these previous solutions to construct the assumed control signals.

(27) $$\begin{array}{c} {\hat{F}}_{u}={\left[{\hat{F}}_{u}\left(0\right),\ldots ,{\hat{F}}_{u}\left(N-1\right)\right]}^{T}={\left[F_{u}^{*}\left(1\right),\ldots ,F_{u}^{*}\left(N-1\right),F_{u}^{*}\left(N-1\right)\right]}^{T}\\ {\hat{F}}_{w}={\left[{\hat{F}}_{w}\left(0\right),\ldots ,{\hat{F}}_{w}\left(N-1\right)\right]}^{T}={\left[F_{w}^{*}\left(1\right),\ldots ,F_{w}^{*}\left(N-1\right),F_{w}^{*}\left(N-1\right)\right]}^{T}\\ {\hat{F}}_{p}={\left[{\hat{F}}_{p}\left(0\right),\ldots ,{\hat{F}}_{p}\left(N-1\right)\right]}^{T}={\left[F_{p}^{*}\left(1\right),\ldots ,F_{p}^{*}\left(N-1\right),F_{p}^{*}\left(N-1\right)\right]}^{T}\\ {\hat{F}}_{q}={\left[{\hat{F}}_{q}\left(0\right),\ldots ,{\hat{F}}_{q}\left(N-1\right)\right]}^{T}={\left[F_{q}^{*}\left(1\right),\ldots ,F_{q}^{*}\left(N-1\right),F_{q}^{*}\left(N-1\right)\right]}^{T}\\ {\hat{F}}_{r}={\left[{\hat{F}}_{r}\left(0\right),\ldots ,{\hat{F}}_{r}\left(N-1\right)\right]}^{T}={\left[F_{r}^{*}\left(1\right),\ldots ,F_{r}^{*}\left(N-1\right),F_{r}^{*}\left(N-1\right)\right]}^{T} \end{array}$$

The presumed state trajectories $\hat{u}\left(s\right),\hat{v}\left(s\right),\hat{w}\left(s\right),\hat{p}\left(s\right),\hat{q}\left(s\right),$ and $\hat{r}\left(s\right)$ can be acquired by progressing through state evolution when using the kinematic Equation (1) and the dynamic Equation (6), thereby commencing from the current time’s updated system state x(t).

The purpose of designing the DMPC algorithm (shown in Algorithm 1) is to simplify the complex trajectory tracking problem for the transformer inspection robot, which involves high dimensionality, multiple subsystems, multiple constraints, and multiple objectives. This algorithm decomposes the complex motion control system optimization problem into several subsystems for solving, thereby significantly reducing the dimensionality and computational complexity of a single optimization problem. The data related to the robot’s state are transferred point-to-point between the five subsystems, and each subsystem solves its optimization problem independently.
**Algorithm 1** Distributed model predictive control (DMPC).1:**if** t=0 **then**2:    Initialization: solve (14) with $x\left(t_{0}\right)=x\left(t\right)$ at t=0, let $u^{*}\left(s\right)$ denote the solution3:**end if**4:**while** t>0 **do**5:    Obtaining system state x(t)6:    Solving subproblems (18), (20), (22), (24), (26) in parallel to obtain the optimal control outputs $u^{*}\left(s\right)$7:    Applying the optimal control outputs over one sampling period8:    The next sampling period, t=t+δ, back to step 59:**end while**

## 5. Simulation of Trajectory Tracking Based on the DMPC

This section presents the simulation results for the transformer inspection robot. These simulations were conducted on a personal computer equipped with an Intel(R) Core (TM) i7-10700 CPU @ 2.90 GHz. The fmincon function provided by MATLAB was employed as the NLP solver for these simulations.

### 5.1. Simulation Setup

The task of a transformer inspection robot is to conduct inspections inside a transformer. This study simulates a robot inspecting around a transformer’s iron core. It is assumed that the iron core has a radius of 0.4 meters and a height of 9 meters. A three-dimensional helix was selected as the desired trajectory, which it can be represented as follows:

(28) $$\left\{\begin{array}{c} x_{r}=0.5\cos\left(0.5t\right)\\ y_{r}=0.5\sin\left(0.5t\right)\\ z_{r}=0.1t \end{array}\right.$$

For the transformer inspection robot, the system parameters were obtained through CFD simulation, where the mass is m=3.249 (kg), the buoyancy is b=3.245 (kg), and the inertial tensor is $I_{0}=\mathrm{diag}\left(0.023,0.030,0.013\right)$ (kg·m^2^). Meanwhile, the thrust limits were $F_{u,\max}=8$ (N), $F_{w,\max}=10$ (N), $F_{p,\max}=5$ (N·m), $F_{q,\max}=8$ (N·m), and $F_{r,\max}=8$ (N·m). In addition, the initial condition was $x={\left[0.5,0,0,0,0,0,0,0,0,0,0,0\right]}^{T}$.

This study uses ANSYS CFX software in combination with the actual conditions inside the transformer to calculate the hydrodynamic parameters of the robot. All hydrodynamic parameters are non-dimensional hydrodynamic coefficients with $X_{\dot{u}}=-33.44$, $X_{u}=19.55$, $X_{|u|u}=-2.45$, $Y_{\dot{v}}=-57.37$, $Y_{v}=17.22$, $Y_{|v|v}=9.31$, $Z_{\dot{w}}=-58.80$, $Z_{w}=39.48$, $Z_{|w|w}=2.59$, $K_{\dot{p}}=-0.061$, $K_{p}=0.2359$, $K_{|p|p}=0.0041$, $M_{\dot{q}}=-0.064$, $M_{q}=0.2435$, $M_{|q|q}=0.0043$, $N_{\dot{r}}=-0.041$, $N_{r}=0.1609$, and $N_{|r|r}=-0.0002$.

For the DMPC parameters, the sampling period was Δt=0.1 (s), the prediction was N=6, and the weighting matrices were $Q=\mathrm{diag}(10^{5},10^{5},10^{5},10^{2},10^{2},10^{2},10,10,10,$10,10,10), $R=\mathrm{diag}(10^{-4},10^{-4},10^{-4},10^{-4},10^{-4},10^{-4})$, and $Q_{f}=\mathrm{diag}\left(10^{3},10^{3},10^{3},10^{2},10^{2},10^{2},10,10,10,10,10,10\right)$.

### 5.2. Tracking Performance and Results Analysis

**Figure 3:**
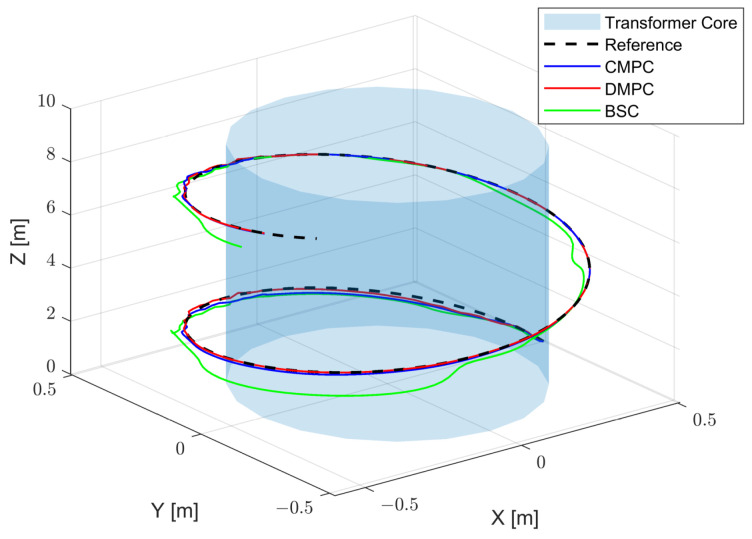
The trajectory under different control algorithms.

The DMPC trajectory tracking control results are presented in Figure 3. In this figure, the black curve represents the reference trajectory, the blue curve represents the simulated robot trajectory under centralized model predictive control (CMPC), the red curve represents the robot trajectory under distributed model predictive control (DMPC), and the green curve represents the robot trajectory under backstepping control (BSC).

**Figure 4:**
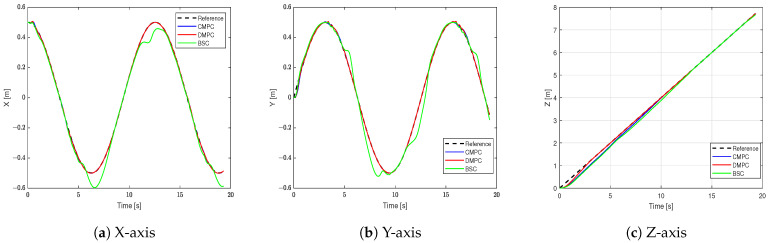
The trajectory in different axes.

Figure 4 shows the tracking results in the X-axis, Y-axis, and Z-axis. It can be seen that BSC can track the set trajectory, but there are noticeable tracking errors in the X-axis and Y-axis. Meanwhile, the CMPC and DMPC closely align with the target trajectory, thus showing excellent tracking performance.

**Figure 5:**
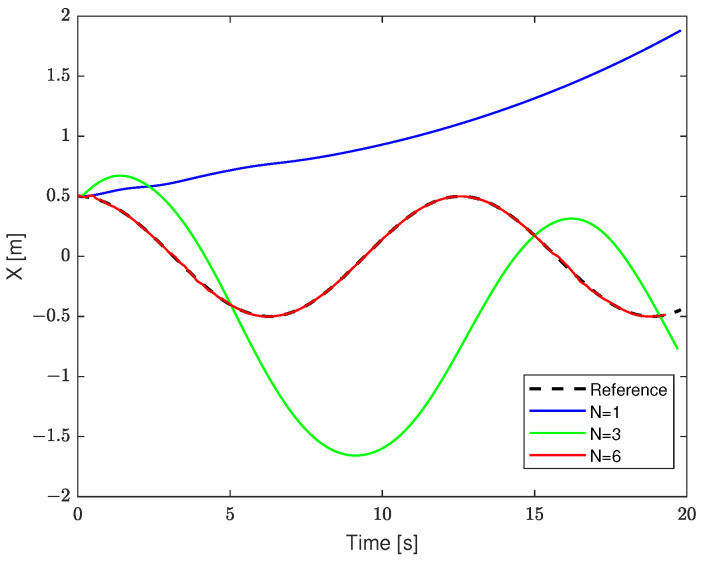
The loss of stability due to a poorly chosen predictive horizon (in the *X*-axis).

Figure 5 demonstrates the loss of stability. If the parameters are poorly chosen, the robot under DMPC control may fail to follow the predefined reference trajectory. As shown in this figure, when choosing the prediction (N=1, N=3), the trajectories will diverge.

**Figure 6:**
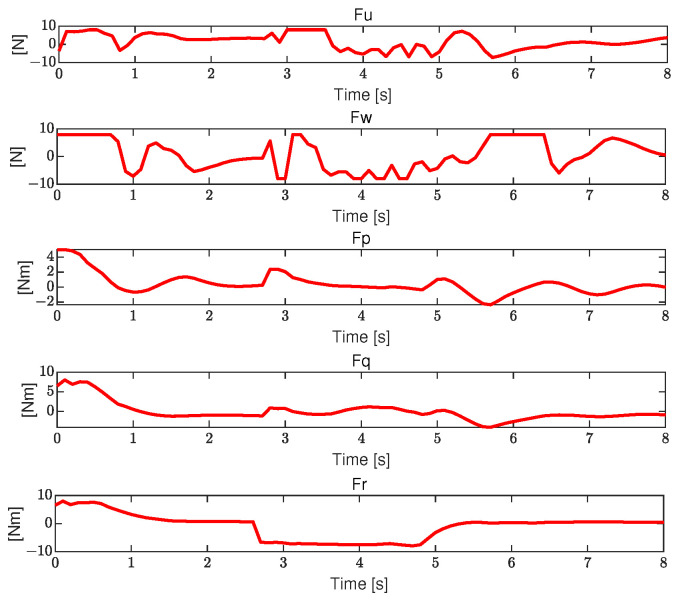
The control forces and moments.

The control forces and moments are demonstrated in Figure 6. As shown in the figure, the control inputs remain good within the allowable limits, thus confirming the efficacy of using DMPC for robot trajectory tracking control.

To compare the computational efficiency of CMPC and DMPC, multiple simulations were carried out, and the average computation time for each of the updates of both methods is listed in Table 2. DMPC has an average update time of 0.0348s, while the CMPC has an average update time of 0.3729 s. Compared to the CMPC method, the proposed DMPC method decouples the centralized system into five subsystems, and it solves the five control variables independently. This significantly reduces the overall dimensionality of the optimization problem for a single update, thereby leading to a 90.66% reduction in computation time.

### 5.3. Robustness Test

In real-world transformer inspection robot applications, disturbances are ubiquitous. In the simulations, the robustness of the tracking control was also investigated. To illustrate the robustness of the CMPC, DMPC, and BSC, simulations of robot tracking control were conducted in the presence of periodic disturbances with $w={\left[5\sin\left(t\right),5\cos\left(t\right),5\sin\left(t\right)\right]}^{T}$.

**Figure 7:**
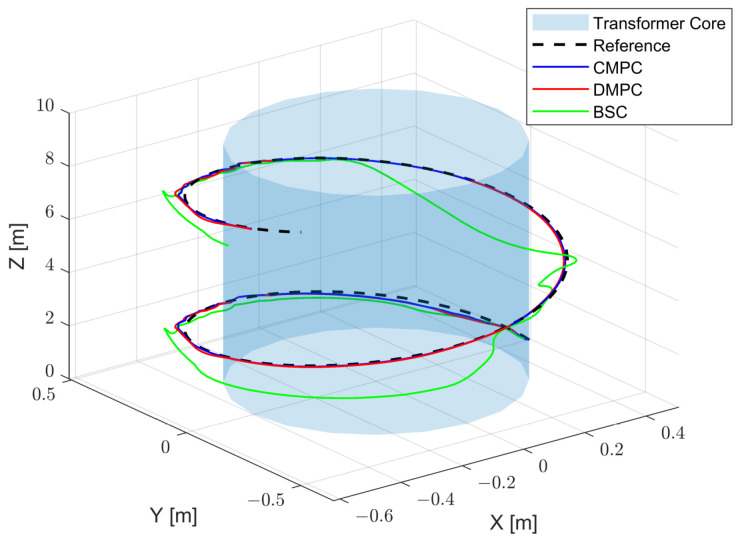
The trajectory with disturbance.

**Figure 8:**
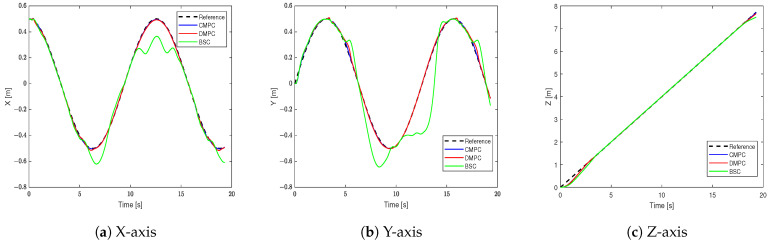
The trajectory in different axes with disturbances.

According to the simulation results in Figure 7 and Figure 8, the CMPC and DMPC tracking control methods successfully guided the robot to converge to the reference trajectory. However, the BSC tracking control method yielded significant tracking errors, so it is not suitable for robot applications.

## 6. Conclusions

This study investigates the problem of trajectory tracking control for transformer inspection robots. Specifically, by designing a distributed model predictive trajectory tracking controller, the robot’s motion is divided into five subsystems, and the nonlinear model predictive optimization problems are solved in parallel. This method effectively addresses the issue of the poor trajectory tracking performance that is caused by the coupling of multiple DOFs, thus leading to improved tracking performance and a significant reduction in computation time due to a large number of control variables. Finally, simulation studies on the robot affirmed the effectiveness of DMPC for tracking control, and they also demonstrated a notable reduction in computation time.

## Figures and Tables

**Table 1:** Thrust magnitude ratio of each thruster in a single-DOF motion.

Moving	T1	T2	T3	T4	T5
Surge	1	1	0	0	0
Sway	0	0	0	0	0
Heave	0	0	−1	−1	$-2L_{2}/L_{1}$
Roll	0	0	$L_{4}$	$-L_{4}$	0
Pitch	0	0	$L_{2}$	$L_{2}$	$-L_{1}$
Yaw	$L_{3}$	$-L_{3}$	0	0	0

**Table 2:** The average computation time per update.

Control Method	Computation Time/s
Centralized MPC	0.3729
Distributed MPC	0.0348

## Data Availability

Data are contained within the article.
